# Benefits and Implications of Resveratrol Supplementation on Microbiota Modulations: A Systematic Review of the Literature

**DOI:** 10.3390/ijms23074027

**Published:** 2022-04-05

**Authors:** Alessio Danilo Inchingolo, Giuseppina Malcangi, Angelo Michele Inchingolo, Fabio Piras, Vito Settanni, Grazia Garofoli, Giulia Palmieri, Sabino Ceci, Assunta Patano, Nicole De Leonardis, Chiara Di Pede, Valentina Montenegro, Daniela Azzollini, Maria Grazia Garibaldi, Zamira Kruti, Antonella Tarullo, Giovanni Coloccia, Antonio Mancini, Biagio Rapone, Alexandra Semjonova, Denisa Hazballa, Maria Teresa D’Oria, Megan Jones, Luigi Macchia, Ioana Roxana Bordea, Antonio Scarano, Felice Lorusso, Gianluca Martino Tartaglia, Cinzia Maspero, Massimo Del Fabbro, Ludovica Nucci, Kenan Ferati, Arberesha Bexheti Ferati, Nicola Brienza, Alberto Corriero, Francesco Inchingolo, Gianna Dipalma

**Affiliations:** 1 Department of Interdisciplinary Medicine, University of Bari “Aldo Moro”, 70121 Bari, Italy; ad.inchingolo@libero.it (A.D.I.); giuseppinamalcangi@libero.it (G.M.); angeloinchingolo@gmail.com (A.M.I.); dott.fabio.piras@gmail.com (F.P.); v.settanni@libero.it (V.S.); graziagarofoli.g@libero.it (G.G.); giuliapalmieri13@gmail.com (G.P.); s.ceci@studenti.uniba.it (S.C.); assuntapatano@gmail.com (A.P.); nicoledeleonardis@outlook.it (N.D.L.); c.dipede1@studenti.uniba.it (C.D.P.); valentinamontenegro@libero.it (V.M.); daniela.azzollini93@gmail.com (D.A.); mr.garibaldi@libero.it (M.G.G.); zamirakruti@hotmail.it (Z.K.); antonella.tarullo@libero.it (A.T.); giovanni.coloccia@gmail.com (G.C.); dr.antonio.mancini@gmail.com (A.M.); biagiorapone79@gmail.com (B.R.); dralexandrasemjonova@libero.it (A.S.); denisahazballa@gmail.com (D.H.); mtdoria51@gmail.com (M.T.D.); megan.jones@live.co.uk (M.J.); francesco.inchingolo@uniba.it (F.I.); giannadipalma@tiscali.it (G.D.); 2 Kongresi Elbasanit, Aqif Pasha, Rruga, 3001 Elbasan, Albania; 3 Department of Medical and Biological Sciences, University of Udine, Via delle Scienze, 206, 33100 Udine, Italy; 4 Department of Emergency and Organ Transplantation (D.E.T.O.), University of Bari “Aldo Moro”, 70121 Bari, Italy; luigi.macchia@uniba.it; 5 Department of Oral Rehabilitation, Faculty of Dentistry, Iuliu Hațieganu University of Medicine and Pharmacy, 400012 Cluj-Napoca, Romania; 6 Department of Innovative Technologies in Medicine and Dentistry, University of Chieti-Pescara, 66100 Chieti, Italy; ascarano@unich.it; 7 Department of Biomedical, Surgical and Dental Sciences, School of Dentistry, University of Milan, 20122 Milan, Italy; gianluca.tartaglia@unimi.it (G.M.T.); cinzia.maspero@unimi.it (C.M.); massimo.delfabbro@unimi.it (M.D.F.); 8 UOC Maxillo-Facial Surgery and Dentistry, Fondazione IRCCS Ca Granda, Ospedale Maggiore Policlinico, 20122 Milan, Italy; 9 IRCCS Orthopedic Institute Galeazzi, 20161 Milan, Italy; 10 Multidisciplinary Department of Medical-Surgical and Dental Specialties, University of Campania Luigi Vanvitelli, Via Luigi de Crecchio, 6, 80138 Naples, Italy; ludovica.nucci@unicampania.it; 11 Faculty of Medical Sciences, University of Tetovo, 1220 Tetovo, North Macedonia; kenan.ferati@apolon.mk (K.F.); arberesha.ferati@unite.edu.mk (A.B.F.); 12 Unit of Anesthesia and Resuscitation, Department of Emergencies and Organ Transplantations, Aldo Moro University, 70124 Bari, Italy; nicola.brienza@uniba.it (N.B.); alberto.corriero@gmail.com (A.C.)

**Keywords:** resveratrol, microbiota, microbiome, nutrition, bone regeneration, immune response, resveratrol supplementation, genic therapy, dentistry, thrombosis

## Abstract

Resveratrol is a polyphenol that has been shown to possess many applications in different fields of medicine. This systematic review has drawn attention to the axis between resveratrol and human microbiota, which plays a key role in maintaining an adequate immune response that can lead to different diseases when compromised. Resveratrol can also be an asset in new technologies, such as gene therapy. PubMed, Cochrane Library, Scopus, Web of Science, and Google Scholar were searched to find papers that matched our topic dating from 1 January 2017 up to 18 January 2022, with English-language restriction using the following Boolean keywords: (“resveratrol” AND “microbio*”). Eighteen studies were included as relevant papers matching the purpose of our investigation. Immune response, prevention of thrombotic complications, microbiota, gene therapy, and bone regeneration were retrieved as the main topics. The analyzed studies mostly involved resveratrol supplementation and its effects on human microbiota by trials in vitro, in vivo, and ex vivo. The beneficial activity of resveratrol is evident by analyzing the changes in the host’s genetic expression and the gastrointestinal microbial community with its administration. The possibility of identifying individual microbial families may allow to tailor therapeutic plans with targeted polyphenolic diets when associated with microbial dysbiosis, such as inflammatory diseases of the gastrointestinal tract, degenerative diseases, tumors, obesity, diabetes, bone tissue regeneration, and metabolic syndrome.

## 1. Introduction

**Figure 1:**
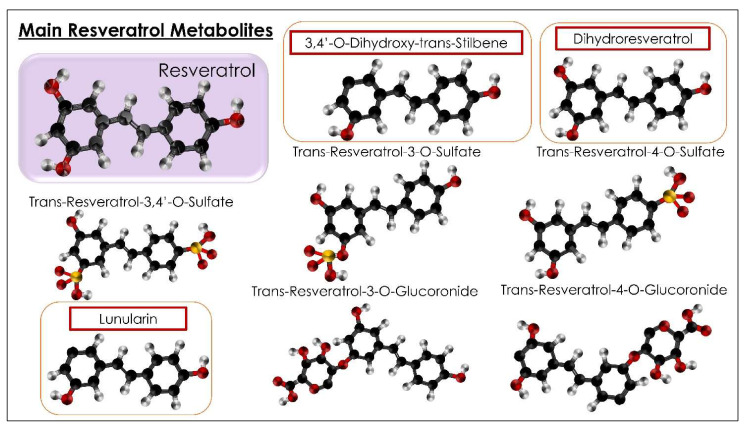
Summary of the RSV metabolites and the main molecules indicated for their antioxidant and anti-inflammatory bioactivity.

Nutrition is one of the most determinative factors of health [1]. Egyptian medicine, one of best-known of the ancient forms of medicine, held nutrition in high account for a purpose: the Egyptian diet was measured and balanced and was considered an art for health and healing. The basis of the diet was: flax, barley, spelt, as well as fruit and vegetables, among which red grapes were especially consumed. Above all, red grapes were widespread in the wealthy classes, who recognized their health and curative benefits [2]. Nutrition’s burden in pathologies, even if not exclusive, is costly for patients in terms of health, expectations, and quality of life. Some aliments (fruits, vegetables) have specific qualities (antioxidants, diuretic, hypoglycemic) in the nutrition sector. These can represent significant effects that implement and develop a high level of human health. One of these is resveratrol (RSV), which is, interestingly, considered as the “molecule of youth” [3,4]. RSV (C_14_H_12_O_3_: 3, 5, 4′-trihydroxy-trans-stilbene) is a powerful natural polyphenol of the phytoalexin family. RSV is present in the barks of some plants, seeds, nuts, peanuts, flowers, and fruits (fermented grapes, mulberry, red wine, blueberries) and in a particular plant called “Japan’s polygon” (Polygonum cuspidatum) [5]. It is found in the form of trans isomer and cis-resveratrol. The first is more stable and has higher bioactivity compared to the second one (six times more potent than cis). Trans-RSV remained stable for several months (protected from the lights and excepted in high pH buffers) [6,7,8]. The higher bioavailability of trans-RSV increases its antioxidant, anti-inflammatory, vasoprotective, anti-mutagenic, anti-proliferative, and anti-carcinogenic actions, making it more effective and longer-lasting [9]. RSV is not soluble in water; however, it is soluble in substances such as ethanol and dimethyl sulfoxide. RSV is known for its anti-inflammatory, antifungal, antioxidant, antithrombotic, anticoagulant, anticancer, and antiviral properties (MERS-CoV, pseudo-rabies, Zika, influenza, Dengue, COVID-19) [10,11,12,13,14,15]. RSV is a polyphenolic sirtuin activator (SIRT-1) that initiates the process of deacetylation of peroxisome proliferator-activated receptor-γ coactivator (PGC-1α), which activates protein phosphatase (PPRy). PPRY takes part in the genomic transcription of those genes that favor mitochondrial metabolism [5,7]. This natural polyphenol takes part in multiple metabolic processes by activating SITR1, which is present in many tissues, such as muscles, pancreas, and adipose tissues [16]. Microbiota (MB) and microbiome (MM) have a substantial difference in meaning (Figure 1) [16,17]. MB indicates all the microorganisms (bacteria, fungi, archaea, protozoa, and viruses) that live and colonize a specific environment in symbiosis, in physiological or pathological conditions. Recent studies estimate the presence of about 38,000 billion bacteria in the human body, mainly living in the intestinal tract. The dominant families (phyla) are *Firmicutes* and *Bacteroidetes* [18,19]. The MM instead indicates the totality of the genetic heritage present in the microbiota and the ability to manifest them [17]. The microbiome study has advanced with metagenomics, which is based on the isolation and genomic sequencing of the 16S rRNA gene, an RNA gene specific to each bacterium responsible for ribosomes production. Identifying these genes means tracing a single bacterial species through a computational analysis of microorganisms, which is also helpful for studying some pathologies dominated by specific families of microbial communities [18,20]. In particular, metagenomics allows us to study the interaction methods of various microorganisms with the environment (microbial ecology) and their precise role in the community [19]. The genome of microbial communities can be studied through a targeted technology: not only sequencing (metagenomics) but also through the transcriptome (transcriptomics), which quantifies the whole RNA that is transcribed by a genome (i.e., microarray technique), and the proteome (proteomics), which studies the whole set of proteins synthesized by mRNA (electrophoretic techniques, chromatographic techniques, mass spectrometry) [21,22]. The metabolome (metabolomics) is another innovative method of studying MM and MB. However, it is not considered an analytical method capable of tracing all the metabolites of a biological microorganism and interacting in metabolic processes [23,24,25]. More and more scientific evidence demonstrates how MB affects metabolic activity and the inflammatory state. MB is recognized as beneficial in compromised psychological states due to its influence on the hypothalamic–pituitary–adrenal axis and the serotoninergic system [23,26,27,28]. The human MB would also intervene in the development of the immune system in the first years of life and in the regulation of the adult immune system [24,29,30]. Eubiosis is mentioned in conditions of equilibrium, dysbiosis in the opposite case. The administration of antibiotics generates alterations in the MB and MM [17,20]. The onset of metabolic, cardiovascular, inflammatory, neurological, psychic, and oncological diseases is often related to dysbiotic conditions [26]. A recent study on the role of the oral microbiome, dysbiosis, and long COVID was performed by metagenomic sequencing using lingual swabs from SARS-CoV-2-infected patients [31,32,33,34,35,36,37,38]. In the oral MB of the patients with long COVID, bacteria such as *Prevotella* and *Villanella* predominated and are known to have inflammatory activity: *Villanella* strains stimulate IL-6 production, while *Prevotella* strains activate IL-23 and IL-1 and toll-like receptor 2 [4,39,40,41,42]. It was also found that the oral MB of patients with chronic asthenia syndrome and myalgic encephalomyelitis was comparable to that of patients with long COVID [43,44,45,46,47,48]. In both, *Leptotrichia*, *Prevotella*, and *Fusobacteria* phyla prevailed, all with inflammatory activities. All the results demonstrate that oral MB dysbiosis may have determined the clinical and symptomatic course of long COVID patients [49,50,51,52]. The latest studies confirm the ability of RSV to interact with intestinal MB and derivatives of their metabolism, such as short-chain fatty acids and intraluminal lipids, playing an essential role in improving the clinical aspects of the metabolic syndrome [16,53,54,55]. In particular, the metabolic syndrome (MS) is a recurrent disease worldwide, characterized by hyperlipidemia, abdominal obesity, insulin resistance, and hypertension, with consequent development of systemic inflammatory diseases, diabetes, coronary heart disease, stroke, and cancer [56,57,58]. Studies show that the oral administration of RSV, activating SIRT-1, influences both glucose metabolism and lipid metabolism, inhibiting their accumulation. RSV inhibits the process of the formation and accumulation of fat in the white adipose tissue, while, in glucose metabolism, it intervenes in several mechanisms [57,59]. Oral administration of RSV in humans improves insulin secretion and insulin resistance by protecting pancreatic β cells from oxidative stress, suppresses glucagon production after meals by improving insulin metabolism, and reduces fasting blood sugar and A1C hemoglobin [60,61]. In the liver, SIRT-1 acts on gluconeogenesis. In tissues where SIRT-1 is present, RSV controls the insulin responses of target cells. Therefore, RSV mimics the effects of a low-calorie diet, which improves cell turnover by slowing down the aging process [62,63,64]. Studies show that there could be an interaction between GM and RSV as if the intestinal microbiota would be the target of RSV, which regulates intestinal homeostasis in response to oxidation processes [65,66,67,68,69]. Most phenolic foods are absorbed in the colon, where they are metabolized by GM into low molecular weight phenolic compounds, such as phenolic acids, which are better absorbed from the intestinal tract to be then conveyed to the liver, where they undergo further biometabolizations (hydrolysis, reductions, and splits) and enter the circulatory system [70,71]. The rate, quantity, and type of metabolites are closely related to the MB variety of the colon. To date, three metabolites of RSV are known: dihydroresveratrol, 3,4′-dihydroxy-trans-stilbene, and 3,4′-dihydroxybibenzyl (lunularin) (Figure 1) [67]. The bioactivity of RSV metabolites could be more intense than RSV itself, and the antioxidant and anti-inflammatory bioactivity of dihydroresveratrol was detected both in vitro and in vivo [72,73,74,75].

**Figure 2:**
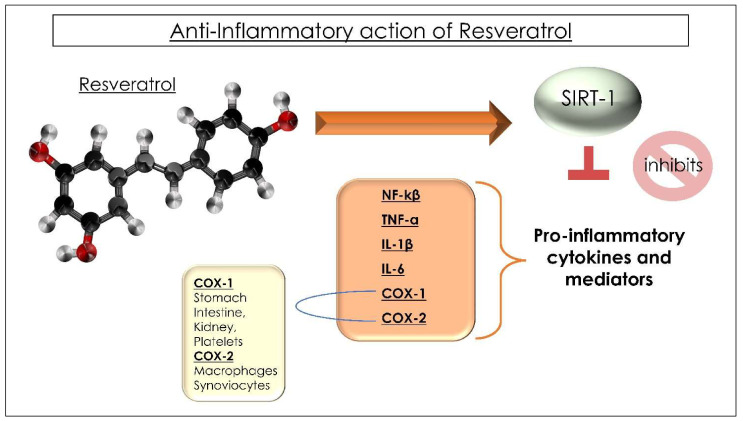
Summary of the cytokines’ and mediators’ modulation correlated to the anti-inflammatory properties of RSV. The RSV is able to interact with the SIRT-1 pathway in order to inhibit the pro-inflammatory mediators’ cascade and the COX-1 and COX-2.

In turn, as for an interdependence relationship, the phenolic derivatives control the GM composition either for or against some microbial strains. Furthermore, it has been observed that some Lactobacilli are proven regulators of the immune system [4], and the qualities of Lactobacilli and Bifidobacteria modulate the gut microbiota. These microorganisms limit gut permeability and favor the immune system [46]. The non-metabolized phenols, together with other endogenous substances of the patient, act as prebiotics influencing the GM with antimicrobial activity and influencing bacterial adhesion to cell surfaces, for example, by favoring strains of Bifidobacterium and Lactobacillus or by inhibiting the growth of different species of Clostridia, *Lachnospiraceae,* and *Enterococcus faecalis* [70,76,77,78,79]. An opposite effect was obtained for *Faecalibacterium prausnitzii*, which was increased compared to the control group. The properties of the prebiotic RSV, which modifies the variability and composition of the intestinal MB, are also manifested in the decrease in the Firmicutes/Bacteroidetes ratio, which instead is increased in obese patients or patients with systemic diseases [80,81,82]. The metabolite dihydroresveratrol (Dh-RSV) comes from the RSV fermentation in the cecum, colon, and rectum by the MB, which acts as a drug in the human large intestine. Therefore, the beneficial effects are not only due to RSV but also due to the products of its metabolism [76,83,84,85,86]. The administration of RSV results in a reduced formation of Trimethylamine-N-oxide (TMAO), a metabolite of carnitine and choline, resulting from the digestion of red meat, egg yolk, and fatty cheeses and liver [87]. A high concentration of TMAO is considered a risk for heart attack and stroke because it activates platelet activity predisposing to thrombosis [87]. Probably, the traditional association that red wine with red meat-based meals acts as a cardiovascular prevention by limiting the production of TMAO has not been observed in white wine consumers or abstainers. Therefore, RSV interacting with the intestinal microbiota could be therapeutic in inflammatory bowel diseases with a systemic implication [81,88,89]. By decreasing the production of interleukin 1 (IL-1), IL-6, C-reactive protein (inflammatory markers), and the transcriptional activity of nuclear factor kappa B (NF-kB), which regulate inflammatory processes and immune responses, RSV reduces inflammation even in patients with cardiovascular diseases, showing improvements in hypertension, heart failure, and ischemic heart disease (Figure 2) [90]. The action of the RSV on the activation of SIRT-1 has also shown benefits on bone metabolism [91].

**Figure 3:**
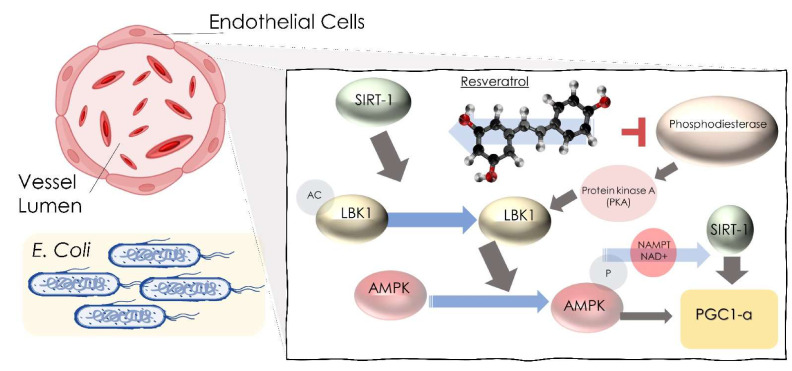
Summary of the pathway correlated to the bacteria infection protective properties of RSV. RSV pathways are able to produce a signals cascade involving Sirtuin 1 [SIRT-1], adenosine monophosphate (AMP)-activated protein Kinase [AMPK] (blue arrow), liver-kinase B1 [LKB1] (grey arrow), and peroxisome proliferator-activated receptor-γ coactivator [PGC-1α].

Some studies show that the concentration of alkaline phosphatase in serum and bone alkaline phosphatase was increased with the administration of RSV, while the values of serum calcium, osteocalcin (a specific marker of bone turnover), and procollagen did not decrease. In a study conducted on type 2 diabetic patients exposed to the risk of fracture, 500 mg/day of RSV was administered, and a reduced loss in bone density was encountered [92,93]. Better results were found in those patients who had lower values of calcium and 25-hydroxy vitamin D and in alcoholic beverage drinkers [91,94]. Some studies have shown how RSV stimulates the activity, and the differentiation processes of osteoblasts slow down the processes of osteoporosis, and, also, combined with platelet concentrates, such as concentrated growth factors (CGF), prevent osteonecrosis from bisphosphonates, in particular from zoledronic acid (ZOL) [95,96,97,98]. At the bone level, the administration of RSV also prevents osteomyelitis due to *Staphylococcus Aureus* by avoiding damage from the neutrophils, caused by the Panton–Valentine leukocidin toxin (PVL), and avoids the development of thrombosis [99,100]. RSV may reduce CNS injury caused by meningitic *E. coli*. In fact, in an in vivo study on mice, it was found that, in the presence of meningitic *Escherichia coli*, the RSV interacting with the lipid raft of the endothelial cells of the blood–brain barrier, blocking the signal cascade of extracellular signal-regulated protein kinase 1/2 and vascular endothelial growth factor-A (ERK1/2-VEGFA), prevented penetration and, therefore, infection of the central nervous system (CNS) and reduced the production of inflammatory cytokines (Figure 3) [101,102]. The antiviral action of RSV is also due to the activation of the intracellular signal of SIRT-1, which blocks viral infections by increasing their resistance [103]. As for COVID-19, studies show that RSV inhibits viral entry into the cell and its replication [4,36,104].

On the other hand, in multiple sclerosis and hepatitis C, RSV administration worsens the clinical outcome [105,106,107]. Recent studies have suggested that RSV improves its stability when combined with glucan. Glucans, also called biological response modifiers, are present in the structure of yeasts, fungi, and algae’s cell walls. For more than half a century, glucan has been recognized as having an active role as a biological immunomodulator [108,109,110,111]. RSV in an aqueous solution with carboxymethyl-β-glucan inhibits the replication of human rhinoviruses (HRVs), responsible for the common cold both in the adult and pediatric population. Precisely, the lack of HRV’s replication seems to be associated with an RSV-induced low cytokine release [108]. Studies show how the use of nasal spray or aerosol with RSV and carboxymethyl-β-glucan has reduced the application of antihistamines and nasal decongestants in the allergic rhinitis common cold, improving symptoms such as cough, rhinorrhea, nasal congestion, sneezing, sore throat, and fever. They were also used as a prophylaxis in pediatric patients exposed to frequent respiratory infections [49,112,113]. An interesting study revealed an inhibitory effect of RSV at the minimum inhibitory concentration (sub-MIC) level on *Streptococcus mutans* and its cariogenic virulence, interfering with the synthesis of acids and their tolerance, on the synthesis of extracellular polysaccharides, on the composition of the biofilm, and on the potential of the virulence gene. For this reason, it could be considered as a product for prophylaxis in the development of dental caries [114,115,116,117]. RSV is considered an antitumor substance since it induces proapoptotic, antiproliferative, anti-inflammatory, and anti-angiogenic mechanisms. One of the main anti-tumor mechanisms of RSV seems to be related to the activation of TANK-binding kinase 1 (TBK1), whose insufficient activity would lead to autoimmune, neurodegenerative, or oncogenic diseases [69,118,119,120]. Many experimental studies recognize the immunomodulatory role and immune function of RSV [79,121,122,123]. RSV modulates innate and acquired immunity by interacting with different cellular targets. However, its properties appear to be conflicting. Its immune functions are dose-dependent: it induces immunosuppression in high doses, while it stimulates the immune system in low doses [124,125]. It intervenes in the immune system by activating macrophages, T cells, and natural killer (Nk) cells with an antioxidant effect, removing reactive oxygen species (ROS), inhibiting cyclooxygenases (Cox), and thus activating anti-inflammatory processes. As an immunomodulator, it inhibits the proliferation of spleen cells stimulated by Concanavaline A and interleukin-2 (Il-2) and blocks T lymphocytes and tumor necrosis factor-α (TNF-α) to produce Il-2 and interferon-gamma (Ifnγ) and macrophages to produce Il-12 [126,127,128]. The RSV bioavailability is rapidly reduced after oral administration, reducing its effects. In fact, traces of RSV are detected in plasma (5 ng/1) after 25 mg administration because 70% is absorbed by intestinal cells, and the remainder is rapidly metabolized [79,129]. As it is not water-soluble, if administered orally, about 30% is directly eliminated without being absorbed [130]. Therefore, to improve the bioavailability, new compounds have been formulated with adjuvants, nanoparticles, phospholipid complexes, and liposomes to evaluate the proper therapeutic dosage in pathological and inflammatory situations and for prophylaxis in physiological conditions [6]. RSV did not show any side effects when administered in the daily dose of 600 mg in chronic diseases and immunomodulatory disorders [104]. Our systematic review focuses on the influence of RSV on the gut microbiota (GM), bone metabolism, and the immune system.

## 2. Materials and Methods

The present systematic review has been performed in accordance to the principles of the PRISMA and International Prospective Register of Systematic Review Registry guidelines (n. 313242) [131]. PubMed, Cochrane Library, Scopus, Web of Science, and Google Scholar were searched to find papers that matched our topic dating from 1 January 2017 up to 18 January 2022, with English-language restriction. The search strategy was built by using a combination of words that matched the purpose of our investigation, whose primary focus is the effect of resveratrol on the microbiota; hence, the following Boolean keywords were used: (“resveratrol” and “microbio*”) (Table 1). Two independent reviewers (A.C., F.I.) working in duplicate evaluated all suitable trials with the following inclusion criteria: (1) studies only on human subjects; (2) open access studies that any other researchers can retrieve without any subscription; (3) studies that analyzed the link between resveratrol supplementation and the effects on the axis microbiota and immune system with a particular focus on oral and intestinal microbiota; studies that did not take into account the microbiota were excluded. Disagreements between the investigators regarding the article’s selection were adequately discussed and resolved by adjusting the inclusion and exclusion criteria.

## 3. Results and Discussion

### 3.1. General Characteristics of the Articles Included

**Figure 4:**
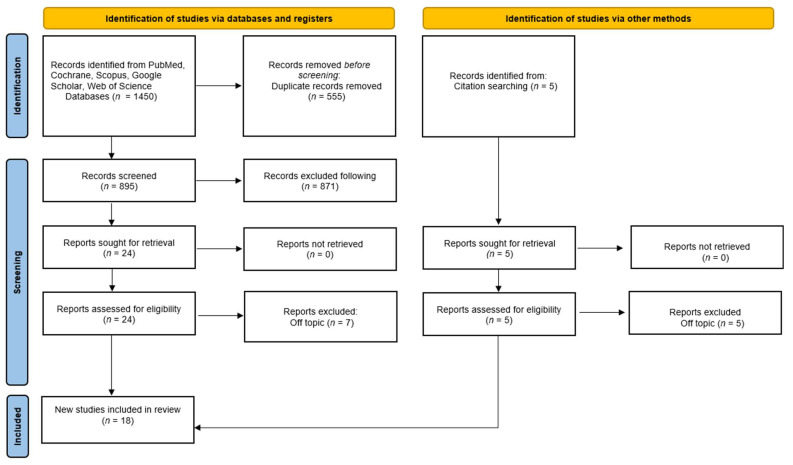
PRISMA flowchart diagram of the inclusion process [131].

A total of 1450 publications were identified from the following databases, including Pubmed (441), Google Scholar (55), Scopus (408), Cochrane (0), and Web of Science (546), which led to 895 articles after removing duplicates (555). Five additional relevant papers were added by searching the reference list of eligible publications. A total of 871 publications were excluded by analysis of the title and abstract. The remaining 24 articles were successfully sought for retrieval and were added to the five papers found by reference list, leading to 29 publications that were assessed for eligibility by the authors. Eleven publications were excluded in the process because they were off-topic. A final number of 18 studies were included in the review for qualitative analysis (Figure 4).

### 3.2. Resveratrol and the Microbiota Modulation

In terms of RSV’s therapeutic potential and benefits, more and more research is finding evidence of RSV’s interaction with the human MM [70,88,132]. Because the totality of bacteria in the human body serves as one of the key regulators in maintaining the homeostasis of many systems in our body [133], alterations in the human MB resulting from RSV usage are also linked to metabolic changes [134]. Metabolic syndrome, which is associated with the development of type 2 diabetes and heart disease, is defined as the presence of three of the following metabolic changes in an individual: obesity or high blood pressure in the central nervous system, high blood glucose, and low serum high-density lipoprotein or high serum triglycerides [55]. RSV has been described as an effective supplement in reducing calories, a process that aids physical activity and insulin sensitivity, leading to increased energy consumption. RSV has also been described as inhibiting adipogenesis, presenting fat-lowering effects [63,135]. These data make RSV a multi-organ/anti-obesity supplement that is worth studying. Walker et al. conducted their study on white subcutaneous adipose tissue. Adipose tissue material obtained for biopsy from both groups was compared before and after RSV treatment, indicating that changes in the expression of genes and expression of gene pathways are inconsiderable; genes related to the mammalian target of rapamycin (mTOR) and SIRT-1 did not change [136]. SIRT-1 is vital in the mitochondria’s function and biogenesis, and repressing PPARγ in adipocytes causes lipolysis and fat loss [135,137]. Nevertheless, RSV in humans has not shown significant effects in weight loss, waist thinning, BMI, or in reducing adipose mass [138,139]. Korsholm et al. conducted their analysis in intracellular pathways, considering these analyses as unbiased and more generalized than the usual tests performed on blood (Table 2).

This biochemical analysis in adipose tissue found considerable increases in intracellular glycerol and free fatty acids in individuals treated with RSV. Six of the thirteen identified lipids experienced a slight increase in individuals of the hRSV group and a slight reduction in dehydroisoandrosterone sulfate (DHEA-S) and 4-androsten-3β, 17β-diol disulfate, which are steroid hormones, leading to the reduction in cholesterol [26]. In MS, a person’s high blood pressure is often present as a consequence of being overweight, and a reduction in cholesterol may bring good results in these cases, but treatment with RSV in humans has not provided satisfactory results either in diastolic or systolic blood pressure [10,141]. The mechanism by which RSV improves insulin sensitivity is already known: RSV activates AMPK [135], so RSV upregulates Act and insulin receptor substrate-1, which are insulin-signaling components [142,143]. RSV reduces the expression of adipokines and adiponectin, which regulate insulin sensitivity and retinol-binding protein 4 expression and resistin [144,145]. However, some studies have not found positive effects of RSV on insulin resistance and glucose homeostasis [138,146,147]. In contrast, other studies acknowledge that RSV does not affect glucose homeostasis and insulin resistance in healthy individuals but in individuals with middle insulin resistance, where it shows a mild action [26,148]. The primary site of postprandial glucose disposal is skeletal muscle [149], and this occurs through the insulin-activated glucose transporter GLUT4 [150,151]. GLUT4 translocation is reduced in insulin-resistant diabetic and prediabetic tissues [152]. Exercise and diet are the strategies that aim to lower postprandial blood glucose in cases of insulin resistance by improving glucose uptake into skeletal muscle cells [153,154]; the MM has an impact on metabolism during exercise [155], and athletes’ GM values differ from those of sedentary people [156]. The impact of the microbial catabolism of phenols on glucose metabolism, particularly in the postprandial period, insulin responses, and type 2 diabetes, was investigated in recent research [157] (Table 3). The effects of certain phenolic compounds on glucose metabolism and absorption by differentiated muscle cells of the human musculoskeletal cell line LHCN-M2 [158], which display typical muscle phenotypic markers, were investigated in detail. The activity of IVAS isovanillic acid-3-O-sulfate, a metabolite of the gut MM, was found to be the most effective in the study [157] (Table 3). IVAS 2 was discovered in plasma after eating berries or cyanidin-3-O-glucoside [159,160], although its biological functions are unknown. According to the findings, IVAS, 3-O-sulfated isovanillic acid enhanced glucose transport as a phase 2 conjugate of protocatechuic acid [161]. Protocatechuic acid acted according to a dose-dependent mechanism in the uptake of (deoxy) glucose in primary human muscle cells [162]. Human adipocytes increased the glucose uptake by mimicking insulin and thereby activating its receptor [163]. Protocatechuic acid and its metabolites may affect both pathways stimulating GLUT4 translocation in L6 myotubes [164]. The mechanism was dependent on PI3K signaling and GLUT4 translocation. IVAS also upregulated GLUT1 and activated Akt (Table 3). According to a study [157] (Table 4), the action of IVAS and IVA isovanillic acid (phenolic conjugate) likely occurs on the insulin receptor present on the surface of LHCN-M2 cells [165], and, because IVATS and insulin are structurally similar, there may be a mechanism of competition between the two molecules that is not yet well understood; this could be investigated in the future using molecular docking and mutagenesis studies. This research demonstrated that conjugated catabolites, derived from the action of the GM, affect glucose uptake and metabolism in LHCN-M2 human skeletal myotubes. Because skeletal muscle is responsible for 75% of postprandial glucose disposal [152], it will be critical to optimize systemic glucose metabolism and muscle function by regulating the insulin-stimulated glucose uptake in muscle cells via the GM [157]. In Caucasian subjects, the post hoc analysis performed 2 h oral GTT speaks of a change in results compared to non-Caucasian (Table 2). Glucose tolerance and insulin sensitivity, according to this study, were notably improved [136]. Using RSV brings about changes in the GM, altering the alpha and beta diversity. Specific taxa changes were also found in persons treated with RSV [136]. Most et al. conducted the study in two groups, polyphenols epigallocatechin-3-gallate (EGCG) and RSV (RES) in the first group and the placebo in the other group. This study compared how the usage of polyphenol supplementation affects the intestinal bacterial flora in both males and females (Table 3). The results showed that taking these supplements considerably reduced *bacteroidetes* in males but not in women (however, this could be due to women’s usage of oral contraceptives, which was not controlled before testing) [132]. According to some studies, polyphenols most likely act on the adhesion of microorganisms and stimulate the growth of commensal bacteria and inhibit pathogenic intestinal bacteria [74,81,166,167]. The selection of bacterial strains depends on their ability to adhere to epithelial cells, production of antimicrobial substances, and survival under simulated gastrointestinal conditions [168]. These characteristics can be studied by in vitro cellular models of the human colon, such as Caco-2, HT 29, T-84, and others. The co-culture of Caco-2/HT29-MTX seeded in the ratio of 9:1 (Caco-2: HT29-MTX) mimics the ratio of Goblet cells to absorptive epithelial cells in the healthy tract [80,167,169], including in the production of mucin. This glycoprotein may have a function in lactobacilli adhesion; in fact, it serves as a rich binding network and substrate for commensal microbiota [170]. Resveratrol improved *Lactobacillus acidophilus* adherence to mucin and HT-29 cells via changes in glycoprotein expression in a recent study [171]; nevertheless, issues remain concerning the physiological significance of the doses utilized in the model. According to the study [172], both *L. gasseri and L. Plantarum* in the presence of RSV adhered with high capacity to the in vitro Caco-2/HT29-MTX co-culture, demonstrating consistency with numerous in vivo *Lactobacillus* studies; these strains colonize the intestinal mucosa after oral administration, and, in particular, *L. gasseri* [173] and *L. Plantarum* [174] were consistently found in the feces. However, on the adhesion of any strain to the physiologically low concentrations of RSV (4.5, 2.25, and 1.125 g mL^−1^) utilized in bacterial suspension, no statistically significant findings (*p* 0.05) were found [172] (Table 4). RSV did not affect the strains examined in the panel, but it cannot be ruled out that it affects other strains. RSV raised Lactobacillus’s adhesion to mucin and HT-29 cells by up to 100 µg mL^−1^ in a recent study [171] despite questions about the relevance of the physiological concentrations used. This suggests that RSV is one of the tested polyphenols that is most efficacious in changing surface protein expression. According to several data, polyphenols have a specific lactobacillus strain and cell line-dependent activity [74]: Epigallocatechin boosted *L. casei* adhesion 3 to Caco-2 cells, while procyanidins B1 and B2 stimulated adhesion to HT-29 cells. Furthermore, epigallocatechin gallate improved *L. acidophilus* adherence to Caco-2 cells [74]. This study demonstrates that, while *L. gausseri* has higher adherence than L. Plantarum, the idea that RSV promotes bacterial adhesion cannot be verified because no statistically significant results were found on the lactobacilli tested [172]. RSV’s favorable effects on the MB and, as a result, on the prevention of noncommunicable illnesses, have only been established in vitro and in rodent models, so more research is required [172]. The binary capability of RSV has been demonstrated in a mice model by Qiao et al. [175], which revealed a selective inhibition of the gut growth of *Enterococcus faecalis* in favor of a growth of *Lactobacillus* and *Bifidobacterium* species. A secondary outcome was associated to an mRNA under-expression of Lpl, Scd1, Ppar-γ, Acc1, and Fas markers that are correlated to the fatty acid synthesis and adipogenesis/lipogenesis processes [175]. Korsholm et al. found changes in the urine, where many of the metabolites derived from aromatic amino acids are the product of MB (Table 2). Metabolites such as tyramine and phenol sulfate, along with homovanillate and tryptamine, as well as indolelactate, 2-hydroxyphenylacetate, and histidine, were changed in the RSV group [26]. Jarosova et al. provided new information on the metabolic process that makes stilbenoids more water-soluble by the human GM depending on their molecular structural properties (Table 4). All this is because the uniformity of stilbenoids varies in the different habitat of the colon. These phenolic compounds have significant biological effects on humans. Many of the most recent nutritional and epidemiological studies confirm these important effects, for example, the ability to defend against oxidative cellular stress and the suitability for the prevention of degenerative diseases affecting the cardiovascular system, neurological system, and even cancer [70]. There are two ways in which resveratrol has affected and modulated the intestinal microbiota: the first with its antimicrobial role, and the second by modifying the composition of this population [176,177]. Its antimicrobial role has been shown to be effective in both Gram-positive and Gram-negative bacteria, for example, the antimicrobial effect of resveratrol on *E. coli* occurs by inhibiting cell division (Z-ring formation) [178,179]. Hydrolysis (O-deglycosylations and ester hydrolysis), cleavage (C-ring cleavage, delactonization, demethylation), and reductions (dehydroxylation and double bond reduction) are the three major catabolic processes in microbial biotransformations [77]. In turn, phenolics appear to influence GM composition by favoring/disfavoring specific microbial strains, establishing a two-way connection between the GM and phenolics [65,81,180,181,182]. Chlorogenic acid, resveratrol, catechin, and some quercetin derivatives, for example, have been shown to increase the proportional representation of Bifidobacterium strains and, hence, have prebiotic-like effects [66,84,183,184,185]. Inoculation with resveratrol and some ellagitannins inhibited the growth of various Clostridia species, demonstrating antimicrobial activity [75,84,181,186]. Bacterial adhesion effects of procyanidin and chlorogenic acid have been observed by Lactobacillus strain adhesion augmentation to intestinal epithelial cells [80,187]. Regarding the composition of gut microbiota, Larrosa et al., concluded that Lactobacillus and Bifidobacterium were increased after the use of resveratrol, and the opposite happened with *E. coli* and Enterobacteria [181]. There is also evidence that confirms the positive role of resveratrol in maintaining gut barrier function and integrity [188]. The changes that resveratrol brings to the composition of gut microbiota are thought to be one of the main mechanisms of how it acts in the body; also, these changes are closely related to the course of metabolic disorders [189,190]. RSV has a strong congeniality with quinone reductase, by the continuous dissociation that reaches a level above 35 nM, so much so that it is possible to define RVS as one of the most potent inhibitors known so far, capable of regulating the cellular presence of antioxidant enzymes and assisting in cellular resistance to stress oxidation [191]. Its anti-inflammatory capacity remains as demonstrated by three pivotal factors: the decrease in tumor necrosis element alpha (TNF-α) and interleukin 1 beta (IL-1ß), amplification of interleukin 10 (IL-10), and reduction in the manifestation of prostaglandin E synthase-1 (PGES-1), cyclooxygenase-2 (COX-2), and inducible nitric oxide synthase (iNOS) [192,193]. RSV affects various metabolic elements and receptors that are responsible for oxidative stress. A study [194] investigating RVS’s effect on intestinal eubiota noted that, with the determination of the internal structure developed by stilbenoids, a reduction in the *Firmicutes* and *Bacteroidetes* ratio was produced, as well as a lowering in the *Clostridium* strains and the incidence in the *Lachnospiraceae* family. The antimicrobial effect of RVS has been demonstrated through its inoculation, which inhibits the growth of several species of *Clostridia* [75,84,181,186]. The total GM is made up of 90% *Firmicutes and Bacteroidetes* [195], and it is known that the increase in F/B in the human (and also mouse) GM is related to an increase in obesity and onset of disease [82,196]. RVS demonstrated to reduce this proportion [84,175,185]. Recent studies lead us to deduce that GM is one of the most important protagonists in cardiovascular diseases, passing through the meta-organismal pathways [197]. In fact, Chen et al. have developed some guidelines for in vitro screening of the mouse gut MM. The results demonstrate that the MB modulates bacterial growth [198]; moreover the cyclic peptides D, L-a of the microbiota are able to reform the intestinal microbioma itself. The GM bases its metabolism on choline, which, by producing trimethylamine (TMA), converts into trimethylamine-N-oxide (TMAO), which implements atherosclerosis by hepatic monooxygenases containing flavin (FMO), and it is known that TMAO has a significant function in cardiovascular disorders [199]. It is noted that TMAO is a product of fish and meat-based nutrition [200] and of the intestinal microbial metabolism of choline, carnitine, and betaine to trimethylamine (TMA). Furthermore, many species of bacteria elaborate that the synthesis of TMA has been developed with cultural methods, as well as those described here belonging to *Firmicutes* and *Proteobacteria*, and *Actinobacteria* [201]. Peptides administered orally daily can reduce the density of total cholesterol, and atherosclerotic plaques and RSV inhibit the synthesis of trimethylamine-N-oxide (TMAO), reducing the growth of TMA with the remodeling of the MM [202]. Intestinal bacteria through choline, carnitine, and TMA synthesize TMAO (Table 4) [203], which has been noted as a cause of cardiovascular disorders.

The whole process is assisted by micro inhibitors that prevent dietary choline or L-carnitine from converting into TMA, which becomes unable to attack the GM. Some researchers confirm that a particular inhibitor, 3,3-dimethyl-1-butanol (DMB), inhibits the microbial activity of choline TMA lyase [204]. In this experiment, *Klebsiella* and *Escherichia* were found to host three of the four potential pathways of production of TMA (choline, carnitine, and TMAO), so they have a significant role in the cycle of TMA in the human intestine (Table 4) [203]. Among the various positive effects of RSV, there are also the anti-cariogenic ones. This activity against *S. Mutans* has been the subject of studies, focusing on acid production, acid tolerance, gene expression, extracellular polysaccharide synthesis, virulence, and biofilm formation [114]. Dental caries is among the most frequent diseases of the oral cavity, and the action of *S. Mutans* mainly causes it. Several antimicrobial agents, including fluoride, have been utilized, but research is moving towards herbal products with minimal side effects. The synthesis of acid and LDH, an enzyme known to create lactic acid during glycolysis, was investigated in a study on the anti-cariogenic features of RSV. *S. Mutans*’ cariogenic activity is achieved through glycolysis and the LDH’s action [205]. According to a study by Li et al. [114], RSV reduces the acid production of *S. Mutans* to sub-MIC levels by suppressing bacterial glycolysis (Table 3). Furthermore, by adding 200 and 400 μg/mL RSV, the final pH values in the glycolytic pH drop test were above the critical pH value that determines the rate of demineralization and remineralization of tooth enamel [206]. RSV reduces the amount of *S. Mutans* at ph 5. Inhibitory activity of RSV on acid tolerance has been demonstrated using proton permeability and F-ATPase activity assays: this action appears to be due to the inhibition of F-ATPase activity, which is essential in maintaining the pH gradient across the membrane, which is related to acid tolerance [207]. The adhesion of *S. Mutans* occurs through water-soluble and insoluble extracellular polysaccharides (EPS), which act in the biofilm matrix’s formation [208,209]. According to the study [114], RSV reduces adhesion and biofilm formation by taking action on soluble and non-soluble polysaccharides, more so on insoluble ones; CLSM images demonstrate a looser and thinner biofilm and thus an inhibitory action on bacterial viability. This study (Table 4) also evaluated the *S.Mutans*’ transcriptional activity and virulence factors, including acid production and acid tolerance, synthesis, and polysaccharide formation. RSV caused lactate dehydrogenase (LDH) gene expression reduction in accordance with LDH activity assay and PCR; RelA gene expression, which encodes for guanosine tetra (penta)-phosphatesynthetase involved in oxidative stress and acid tolerance mechanisms [210], was also decreased. The activity of the gtfC gene encoding for GTCF, which is required for glucan synthesis in the biofilm, was also discovered to be altered, resulting in lower cariogenic activity in vivo [211,212]. RSV can repress the ComDE system of *S. Mutans,* generating a phenotype with a defective biofilm [213]. The ComDE system is a fundamental quorum, sensing the cell–cell communication system in the gene regulatory networks responsible for bacterial adaptation in biofilms [115,214]. Repression of this gene interferes with the internal communication quorum sensing mechanism in *S. Mutans* by inhibiting biofilm formation. According to this study, RSV might have anti-cariogenic effects, but the toxic effect in the oral cavity is still to be elucidated, so further studies are needed to understand its molecular mechanism.

### 3.3. Resveratrol and Microbiota Modulation on the Immune Response

The MB invades and occupies the intestine from birth and is immediately conditioned by the type of birth, breastfeeding, and antibiotic intake [216]. The MB is the set of symbiotic microorganisms in symbiosis with the human body, and it is strongly regulated by food and microorganisms that regulate the bioavailability of many antioxidants [217]. Being in direct contact with the outside world, the oral MB is the first point of contact with everything we ingest. It has a defensive role of primary importance and acts as a sentinel against potential bacteria and viruses that can infiltrate and try to reach the respiratory tract, promoting infection [218]. The microbiota is a variable organ, modified by hormonal factors specific to the person or external factors, such as diet or probiotics [46,219]. However, if there is dysbiosis, the bacteria present are mostly pathological, and the “line of defense” is not strong enough to protect us from possible pathologies [220]. Pathogenic viruses and bacteria can prevail and inflame the tonsils, pharynx, and larynx, leading to pharyngotonsillitis, pharyngitis, tonsillitis, or halitosis oral candida [221]. Polyphenols can be an excellent help for available diseases, weight, and metabolism, helping to increase the risk for different diseases [222]. Plants synthesize RSV as one of the defense mechanisms, which regulates immunity and can play a role in the prevention and evolution of chronic inflammatory diseases as it interferes with the regulation of immune cells, the synthesis of pro-inflammatory cytokines, and gene expression [90]. RSV can directly damage bacterial, fungal, and viral cells, hitting specific targets within these microorganisms and slowing their growth [223]. RSV has antimicrobial activity: it is capable of recruiting and activating macrophages, neutrophils, and lymphocytes during infections [121]. It has an anti-inflammatory action, stimulating that of anti-inflammatory cytokines, reducing chronic inflammation: by activating SIRT-1, it inhibits the formation of inflammatory factors, which include NF-kB, which increases the production by the cells of the immune system of pro-inflammatory cytokines (such as TNF-α, IL-1β, IL-6) and COX (COX-1 and 2), which transforms arachidonic acid into inflammatory prostaglandins and thromboxanes. The anti-inflammatory function of RSV is mediated by SIRT-1 [121]. RSV also reduces oxidative stress and increases antioxidant molecules. These molecules, reducing the production of free radicals, are the inhibiting glutathione S-transferase (GST), glutathione peroxidase (Gpx), NQO1, catalase, and superoxide dismutase (SOD) [224]. Ramdani [225] demonstrated that RSV regulates RAS and ACE2, involved in SARS-CoV-2 disease, reducing inflammatory cytokines, activating the SIRT-1 and p53 signaling ways, and by increasing CTL and NK cells. The receptor for SARS-CoV-2 is the angiotensin-converting enzyme 2 (ACE-2). Inhibition of ACE reduces cardiovascular and renal diseases. Furthermore, ACE-2 can reduce the binding of the SARS-CoV 2 virus to human cells. RSV works by inhibiting ACE/ACE-2. RSV increases the activity of the angiotensin II converting enzyme receptor both in vivo and in vitro [226]. Cells with high ACE2 expression on their cell walls are an easy target for the SARS-CoV-2 virus [4]. RSV would have shown the potential to increase the expression of ACE-2, important in the role of the entry of SARS-CoV-2, and could intervene in the SARS-CoV-2 infection [227]. Research tells us that RSV is associated with carboxymethyl beta-glucan (CMG), which increases its solubility and blocks the SARS-CoV-2 virus replication in human nasal epithelial cells [228]. RSV could play an important role in the regulation of the renin–angiotensin system (RAS) and activation of ACE 2 [229]. RSV works by activating sirtuin 1 (SIRT-1) [230], which has a protective role in response to stress, inflammation, and the regulation of apoptosis [231]. The activation of SIRT-1 and superoxide dismutase (SOD) of RSV is associated with increased ACE2 function and decreased markers of inflammation. Therefore, the upregulation of ACE by the RSV could play an essential role in SARS [232]. RSV modulates inflammatory components and exercises immunoregulatory effects. The red wine polyphenols have a positive effect on bacteria, which cause problems with teeth and gums. The 2% RSV emulgel is very effective in improving gum health in orthodontics, reducing gingival inflammation [233]. In particular, RSV has inhibitory properties on the cariogenic virulence of Streptococcus mutans, reducing acid production and biofilm formation. The expression of the related virulence gene was also downregulated with increasing RSV concentrations. RSV, therefore, represents a promising anti-cariogenic agent, reducing the ability of bacteria to stick to teeth and gums [114]. In addition, there are studies on periodontitis, particularly on the inflammatory responses induced by *Porphyromonas gingivalis* in human gingival fibroblasts [234]. The mechanism by which RSV can increase antioxidant enzymes in our body is the activation of the *Nrf-2* gene, involved in the synthesis of the antioxidant molecules listed above [235]. RSV is considered to be more potent than vitamin E, one of the most potent antioxidants known to date [236].

Liu et al. [237] (Table 5) analyzed antimicrobial peptides (AMP: α- and β-defensins, cathelicidin LL-37, and statins 5), which have antimicrobial and immunostimulant properties, an important defense mechanism of the innate immune system against invading microorganisms. The bactericidal activities of AMP are due to the formation of pores in bacterial cytoplasmic membranes. Furthermore, having a distinctly positive charge, AMPs enhance the initial electrostatic attraction to negatively charged lipid membranes and negatively charged acids on the surface of Gram-positive bacteria, for example, *Staphylococcus aureus*, a common opportunistic human colonizer that may cause life-threatening diseases, such as sepsis, endocarditis, and pneumonia. The sensitization of *S. aureus* to AMPs of the innate immune system can facilitate the eradication of *S. aureus*. RSV exhibits antibacterial effects and is a presumed inhibitor of ATP synthase in *S. aureus*. This indicates that ATP synthase inhibition can be used to sensitize S. aureus to the natural AMps of the innate immune system. The ATP synthase inhibitor is RSV. Inhibitors can be adjuvants for antibiotics [223]. The mosquito-borne (*Aedes* mosquitoes) disease caused by a flavivirus is DENV, and it is endemic to tropical climate areas of the world. DENV fever [238] is generally high and sudden, characterized by headache, myalgia, arthralgia, generalized lymphadenopathy, and rash after a brief apyrexia, and, in addition, coughing, sore throat, and runny nose. DENV can also cause fatal fever with bleeding diathesis and shock. Zainal et al. demonstrate that RSV has antiviral activity against DENV. RSV suppresses DENV replication by inhibiting the translocation of the high-mobility group box 1 (HMGB1), a DNA-binding protein from the nucleus. The RSV inhibits this migration and increases the interferon-stimulated genes’ (ISG) transcription by nuclear HMGB1 (Table 5). RSV inhibits the translocation of HMGB1 out of the nucleus, allowing pro-inflammatory genes to be downregulated during DENV infection [14]. Yang [101] examined the action of RSV in inhibiting meningitic invasion of meningitic *E. coli* in the blood–brain barrier (BBB). RSV inhibits bacterial penetration of BBB, reduces neuroinflammation and lethality, reducing the inflammatory cytokines and the upregulation of caveolin-1 (CAV-1), a class of rafts that is supposed to function in endothelial cells. RSV may prove to be important in the inhibition and management of *E. coli* meningitis and may alleviate *E. coli*-induced meningitic CNS injury (Table 2). Hwang and Lim [239] found that, in the Escherichia coli, is the AcrAB-TolC multi-drug efflux pump (Table 5). The *E. coli* AcrAB-TolC complex often transports toxic compounds out of the cells, and this pump has importance in drug resistance: in cases where there are high AcrAB levels, they are measured in the cases of multi-resistance of antibiotic-resistant strains, such as MRSA (methicillin-resistant Staphylococcus aureus). Efflux pump inhibitors (EPIs) are interesting molecules to test as potential adjuvants for antibiotic treatment because they hinder multidrug resistance in *E. coli*. The key goal of RSV is to stop bacterial growth. RSV significantly cut the activity of the AcrAB-TolC drug efflux complex, promoting the antibacterial activity of RSV in *E. coli* [239]. Cold is a communal viral infection (*rhinoviruses (HRVs)*) that causes inflammation of the nasal mucosa and pharynx (throat). Both sick and healthy carriers can transmit highly contagious colds. The common cold has mild symptoms but can be severe in children. According to the results of Baldassarre et al. [108], the solution with RSV plus carboxymethyl-β-glucan in the treatment of childhood colds reduces respiratory symptoms and relapses (significant reduction in sternutation and episodes of productive or non-productive cough). In children, RSV with CM-glucan can alleviate nasal problems and breathing difficulties, for example, allergic rhinitis and acute nasopharyngitis (Table 2). Based on the information mentioned in the above articles, having an intestinal microbiota composed of symbiotic microorganisms in balance with the immune system is of fundamental importance in order to be able to carry out an effective defense against external pathogens to which one may be exposed. Conversely, a condition of imbalance of the microbiota and of our immune system leads to a dysbiosis that negatively predisposes both innate and acquired immune defense, compromising it. Therefore, by virtue of the prebiotic role of RSV and the role of the stimulator of the immune system, it is possible to affirm its usefulness as a preventive and adjuvant factor of traditional antibiotic therapies against many diseases caused by pathogens. For the reasons we have analyzed, it can be said that RSV can undoubtedly be considered an excellent supplement to our everyday eating habits. The results derived from immunological research have undoubtedly generated increasing enthusiasm as RSV displays remarkable antibacterial and antiviral properties, which is why it will be necessary to continue the study on RSV given that it keeps our immune system in a state of alert activity and influences the intestinal microbiota (Table 5).

### 3.4. Resveratrol and Microbiota Implications on Preventing Thrombotic Complications

Regarding the protective effect of RSV, a natural polymeric compound contained in grapes, it has antibacterial mechanisms against *Staphylococcus aureus (S. aureus*), reducing the inflammatory response in infected tissues [99]. RSV is a potentially useful agent in preventing thrombotic infections caused by the *S. aureus* strains responsible for major infectious diseases, such as osteomyelitis [212]. *S. aureus* is a GRAM+ bacterium with a spherical and aerobic shape that gives rise to colonies in which the microorganisms form a cluster arrangement. It is a very virulent bacterium capable of causing several diseases. It usually colonizes the skin and mucous membranes, and an effective immune system can keep the microorganism under control. However, in immune deficiency, it can spread through the bloodstream and affect joints and bones by establishing osteomyelitis. *S. aureus* induces an inflammatory reaction in the host, leading to the excessive and rapid recruitment of inflammatory cells [240]. Following the lysis of neutrophils, which are found to be the target of PVL toxins produced by *S. aureus*, pro-inflammatory substances such as IL-6, IL-8, and TNF-α are released. These are responsible for thrombus formation when associated with osteomyelitis [241,242]. PVL is a toxin associated with the development of thrombosis in patients with osteomyelitis. It is made of two parts (LukS-PV and LukF-PV) that create beta-barrel pores. The lukS-PV component binds to the complement receptor C5aR, and both components then result in the insertion of the hydrophobic stalk into the target cell membrane. Once damaged by PVL, Neutrophils release prothrombotic cytokines, antimicrobial alpha-defensins (HPNs), myeloperoxidase (HOCL), and myeloperoxidase-modified proteins. RSV can inhibit these substances from damaged neutrophils [243,244]. Thrombotic problems can occur in those with a weakened immune system, and high levels of PVL can be seen in the context of bone infections [100]. The toxin secreted by *S. aureus* plays a central role in the development of thrombosis due to the interaction between PVL toxin, neutrophils, and platelets [99]. PVL induces neutrophil lysis and the release of HPNs and HOCL [100,245,246]. HPNs activate platelets [247], accompanied by the conformational change in the platelet fibrinogen receptor GPIIb/IIIa, which increases its affinity, inducing the formation of microparticles that induce thrombin generation and thus are essential in thrombus development [248]. There is a two-step mechanism in the development of osteomyelitis produced by *Staphylococcus aureus* that is determined by the activity of PVLs on platelets: A high number of neutrophils accumulate at the site of osteomyelitis, which is lysed by PVLs, inducing them to release HNPs or myeloperoxidase and subsequently activating platelets either directly or through lysine-presenting proteins. This mechanism causes thrombosis through platelet aggregation [248]. RSV can counteract the platelet activation effects of HOCL-modified proteins and inhibit neutrophil myeloperoxidase. Moreover, it blocks PVL-induced fibrinogen-platelet binding. RSV protects vessels from plaque formation and proliferation of smooth muscle tissue, stimulates nitric oxide production responsible for vasodilation, and inhibits platelet activation and aggregation, the first step in blood clot formation [249]. As a result, *S. aureus* is one of the microorganisms that might cause life-threatening complications. It is important to prevent its virulence. *S. aureus* has long been suspected of having its primary reservoir in the nasal cavity [250]. Recent studies, however, have shown that *S. aureus* is part of the oral MB in greater quantity than the nasal one [251]. *S. aureus*, in a 2008 study, was also found to be common in the oral cavity, with the prevalence ranging from approximately 33% in dental plaque to 47% in saliva [252]. In addition, it is recognized that the use of dental prostheses numerically elevates oral pathogens. In these cases, proper oral hygiene is recommended to avoid the establishment of distant or local infections [78,251]. Some oral diseases are caused by this microorganism, e.g., angular cheilitis, mumps, staphylococcal mucositis, failure of dental implants [253]. MRSA species have also been isolated in the oral cavity. These species are difficult to treat with classical antibiotics because of their resistance. They are very dangerous because they can have the gene for PVL, causing the previously reported disruptions [254]. Based on the information mentioned, we firmly believe that the oral condition of *S. aureus* colonization must be maintained at nonvirulent levels to avoid the establishment of the described diseases. RSV, as pointed out, aids in the maintenance of the oral MB.

### 3.5. Resveratrol and Microbiota Implications in Gene Therapy

Given its remarkable and important biochemical characteristics, in recent years, RSV has also been studied as an adjuvant in cutting-edge technology. Gene therapy is a modern approach that involves the manipulation of genetic material to treat specific diseases. This manipulation allows replacing a defective or mutated gene with a valid one or modifying an existing gene to change its function [255]. This technique involves modifying the gene responsible for the genetic disease and inserting it into a DNA vector. The vectors most commonly used to transport genetic material are viral vectors due to their efficiency in invading cells and introducing their genetic material [256]. The rationale for the procedure is to get the vector to transport the functioning gene into the target cell and have it integrated into the DNA to treat the disease. The inserted foreign genetic material must be stable in the host cell in order for the therapy to be successful [257]. This technique has proven efficacy in treating several hematological disorders using hemopoietic stem cells as target cells [258,259]. Transduction resistance of hematopoietic stem cells is the major problem of lentiviral vector-mediated gene therapy [260], previously attributed to inhibition by the proteasome, the absence of vector receptor [261], or a cell in a quiescence state [262]. The interferon-induced transmembrane (IFITM) family of proteins also have intrinsic defensive effects against pathogens that use cellular endosomes for entry and transport. In particular, IFITM3 limits the gene delivery efficiency mediated by vesicular stomatitis virus (VSV) in hematopoietic stem cells [263]. Further, mTOR inhibitor rapamycin can block this process pharmacologically, but this results in numerous immunosuppressive side effects [264,265]. A study by Ozog et al. proposed to investigate RSV as a viable component to enhance gene transport in hematopoietic stem cells [266]. Therefore, in this study, RSV and some of its synthetic cyclic compounds were examined to enhance the lentiviral vector-mediated gene delivery in this type of cells. The results showed that only a synthetic compound, CaraphenolA, a synthetic RSV cyclotrimer of a higher oxidation state, was able to enhance gene delivery [266]. Specifically, Caraphenol A substantially improved gene delivery through a mechanism that resolves the impediment by the IFITM2/3 to the vector. Notably, the modulation by this compound did not result in unusual integration or lineage disorders in vivo [266]. In addition to being a good asset in bioengineering [266], RSV can be used as an adjuvant in the treatment of hematological cancer disorders and other tumors [267,268]. In fact, several studies have shown that its anti-tumor activity acts on several levels. It indirectly decreases oxidative and inflammatory stress by minimizing reactive oxygen species, inhibits the phase I cytochrome P450 enzymes responsible for carcinogen activation, induces the death of malignant cells, and suppresses the proinflammatory signaling pathways linked to cancer progression [118,120,267,269]. Moreover, patients with cancerous hematological disorders are significantly immunosuppressed due to chemotherapies that encourage the development of opportunistic infections linked to alterations in the MB that make it pathogenic [270,271]. In addition, individuals treated with hematopoietic cell transplantation (HCT) may develop graft-versus-host disease (GvHD), which severely attacks the gut. Bacteria, particularly the GM, have recently been recognized as important in the success of HCT and the onset of GvHD [272]. In particular, the lack of MB is a risk factor for treatment-related mortality, including death from GvHD, infection, opportunistic illness, and organ failure after HCT [273]. Therefore, with the mechanisms previously discussed in this paper, we suspect that RSV may also have a role in controlling the GM in this cancer scenario.

### 3.6. Resveratrol and Microbiota Implications in Bone Regeneration

Based on the literature, RSV can promote the differentiation of bone cells and the release of angiogenic factors that result in better bone vascularization with a greater supply of nutrients and growth factors. This leads to bone formation [274,275]. Zhang et al. [276] showed that RSV associated with strontium ranelate (SrRn) induces osteogenic differentiation of mesenchymal stem cells (MSCc). This aspect was deduced by an increase in alkaline phosphatase (ALP) activity with an increase in the expression of the transcription factor runt-related transcription factor 2 (RUNX-2), osteocalcin (OCN), and collagen 1A1 (Col 1A1). Other studies report that RSV influences the osteogenic differentiation of MSCs because bone morphogenic protein-2 (BMP-2) activates SIRT-1. The latter increases the activity of ALP and human osteoprotegerin (OPG) [274,277,278]. The Notch signaling pathway, which RSV activates, favors the conversion of osteoblasts into osteocytes [279]. Furthermore, in the study of Zhang et al., RSV increases the expression of the nuclear factor of activated T cells 1, which is present (NFATc1; transcription factor for the differentiation of osteoclasts [280]); RSV has no effect on the expression of Matrix Metalloproteinase-9 (MMP-9; metalloproteinase involved in the destruction of the extracellular [281]) and Cathepsin K (CTSK; protease involved in the degradation of collagen and in the bone resorption phase [282]) [276]. Some studies show that RSV inhibits the action of osteoclasts by activating the Wnt/β–catenin pathway [283,284]. Several studies state that RSV causes an increase in vascularization and, consequently, bone formation [275,276]. In the study included in our review, the effect of RSV on human umbilical vein endothelial cells (HUVEC) was evaluated. It was noted that RSV promoted angiogenesis by increasing VEGFA, platelet–endothelial cell adhesion molecule (PECAM), and von Willebrand factor (vWF) [276].

In the work of Zhang et al. [276], the following aspects were analyzed:

1. the differentiating power of the RSV associated or not with SrRn on the MSCs;
2. the inhibitory effect of RSV on osteoclasts;
3. the angiogenesis effect of RSV associated or not with SrRn on HUVEC;
4. Formation of bone in rats. Bone defects were induced on the mandible of rats and then rehabilitated with 3D scaffolds (loaded with a mixture of RSV, SrRn, or both). Four groups of rats were evaluated: rats with scaffold only, rats with scaffold + RVS, rats with scaffold + SrRn, and rats with scaffold + (RVS + SrRn).

The results of this study show that [276]:

1. MSCs proliferate and differentiate with a high rate on scaffolds with SrRn, and the rate is higher than that on scaffolds alone and on RVS scaffolds;
2. on the scaffolds with RVS, SrRn, or both, there was a reduction in the number and size of osteoclasts compared to that observed in the group with only the scaffold;
3. RVS scaffolds have a more significant angiogenesis effect than SrRn or scaffold-only groups;
4. The micro-computed tomography (CT) analysis showed high bone formation in rats in which scaffolds with RVS and SrRn were implanted compared to the other two groups.

Another study [95] evaluates, through the in vitro analysis, the action of RSV associated with CGF on the differentiation and proliferation of human osteoblasts. Furthermore, the protection of RSV on osteoblastic cells treated with bisphosphonates was evaluated. The bisphosphonates administered on osteoblastic cells in vitro are Alendronate (AL) and ZOL. These drugs are administered in diseases, i.e., osteoporosis, metastatic bone cancer, and Paget’s disease. Bone regeneration can be compromised by prolonged use of these drugs, resulting in necrosis of the maxillary and mandibular bone tissue (osteonecrosis) [285,286,287].

In the study by Borsani et al. [95], the control group is represented by human osteoblastic cells in osteoblast growth medium. This study highlighted that:

1. CGF and RSV (10 μM) have an osteogenic effect and protect osteoblasts treated with ZOL;
2. OPG levels are found to be elevated in osteoblasts treated with RSV (10 μM) and ZOL or CGF associated with RSV (10 μM) and ZOL. Meanwhile, in the treatment with AL, there is no increase in OPG;
3. the treatment with CGF, RSV (10 μM), and AL or CGF, RSV (10 μM), and ZOL resulted in a significant increase in BMP-2 (inducer of osteogenesis [288]) levels but less than in osteoblasts treated with CGF and ZOL;
4. in osteoblastic cells treated with RSV, CGF, and ZOL, there is an increase in SIRT-1 and Col 1A1;
5. human osteoblastic cells treated with RSV (10 μM) deposited significant amounts of calcium, unlike the control group (human osteoblast cells in osteoblast mineralization medium).

Numerous literature studies state that the influence of RSV on osteogenic differentiation, proliferation of human osteoblasts, and endothelial cells depends on the dose of the drug [289,290,291].

In the study by Zhang et al. [276], the concentration of RSV released from the scaffolds was equal to 2.15 ± 0.16 µg/mL, while, in the study by Borsani et al. [95], it is stated that the concentration that determines an increase in the proliferation of osteoblasts without cytotoxic effects is equal to 10 µM.

By adopting a concentration of RSV equal to 25 μM, Ornstrup et al. [292] observed that this stilbenoid has an osteogenic action but reduces the proliferation of MSCs. Furthermore, different authors [289,290,293] described that, for a concentration of RSV between 0.1 μM and 2.5 μM, there is an increase in the proliferation of MSCs deriving from the human umbilical cord (hUC-MSCs), while proliferation is reduced to concentrations between 5 μM and 10 μM. Finally, high concentrations of RSV inhibit angiogenesis and HUVEC migration [294]. Our analysis shows that RSV has an osteogenic action as it induces the multiplication and differentiation of MSCs and plays an essential role in angiogenesis given by the proliferation of HUVEC. The action of RSV seems to be empowered by SrRn as the latter should play a key role in inhibiting osteoclastogenesis. This leads to a reduction in bone reabsorption in favor of bone deposition. Furthermore, RSV protects human osteoblasts treated with bisphosphonates. This appears to be potentiated by CGF, which contains autologous platelet-derived osteoinductive growth factors. Thus, the RSV appears to modulate the effects of the CGF. Table 6 summarizes the results obtained.

Numerous studies have shown that RSV increases the expression levels of SIRT-1 [274,295,296] even in rats in which an ovariectomy has been performed. This leads to thinking about the positive effect of RSV in the case of post-menopausal osteoporosis [297,298,299,300]. Therefore, RSV has been shown to activate SIRT-1, causing osteogenic MSCs differentiation and proliferation [274,277,301]. This leads to bone formation [298,302,303]. RSV also promotes the conversion of osteoblasts to osteocytes via the Notch pathway [279]. RSV appears to have a protective effect against osteoblastic cells treated in vitro with bisphosphonates [95], and the study by Zhai et al. demonstrates that RSV prevents steroid-induced osteonecrosis in a group of rabbits [96].

Other studies show that RSV inhibits the action of osteoclasts by activating the Wnt/β–catenin pathway [283,304]. Several studies state that RSV causes an increase in vascularization and, therefore, bone formation [275,302,305]. RSV increases osteoblast proliferation while inhibiting osteoclast differentiation [306,307], making it a promising candidate for research in dentistry and maxillofacial surgery [95,276,308,309,310,311]. Due to the minimal capacity to trigger an immune response, bone regeneration is currently carried out with autologous transplants [312]. The utilization of MSCs is a novel strategy, but it has a critical drawback: harvesting difficulty and poor long-term stability. Another option is to employ biomaterials; however, the clinical guidelines for doing so are currently unknown [313]. RSV, which is isolated from natural sources, triggers pathways that lead to osteoblastic development and differentiation [95,276,314]. As a result, administering RSV could be a viable therapeutic option for the processes of bone repair and defect restoration [315]. As demonstrated in the study by Zhang et al. [276], using RSV alone or combined with scaffolds can improve the clinical management of diseases such as osteonecrosis of the jaw caused by long-term bisphosphonate medication.

## 4. Conclusions

RSV has long been recognized for its anti-inflammatory, antifungal, antioxidant, antithrombotic, anticoagulant, antitumor, antiviral, and immune properties. Therefore, it can bring multiple beneficial effects to the body as it plays an essential role in preventing many pathologies by positively modulating the human GM. Its anti-inflammatory action is due to the activation of SIRT-1, which inhibits the formation of inflammatory factors, such as NF-kB, which stimulates the immune system to produce pro-inflammatory cytokines (such as TNF-α, IL-1β, IL-6) and cyclooxygenases (COX-1 and 2), thus reducing oxidative stress. In addition, the activation of the Nrf-2 gene, which is involved in synthesizing antioxidant molecules, enhances its antiphlogistic abilities. During an infectious process, RSV exerts its antimicrobial activity by recruiting and activating macrophages, neutrophils, and lymphocytes. In the oral cavity, RSV has inhibitory properties on the karyogenic virulence of Streptococcus mutans, reducing acid production and biofilm formation. Used as a nasal spray or aerosol, RSV combined with carboxymethyl-β-glucan has reduced the use of antihistamines and nasal decongestants in allergic rhinitis and the common cold, improving symptoms, such as cough, rhinorrhea, nasal congestion, sneezing, sore throat, and fever. At the level of bone metabolism, the study shows that RSV, by activating SIRT-1, promotes osteogenic proliferation by the differentiation of MSCs and angiogenetic proliferation because it stimulates the proliferation and migration of HUVECs. RSV associated with SrRn inhibits osteoclastogenesis by reducing bone resorption and promoting bone synthesis. RSV associated with CGF enhances its protective effects against osteonecrosis of the jaws, induced by taking bisphosphonates, by promoting osteoinductive processes and angiogenesis. Osteoblastic and angiogenetic activation are closely related to low doses of RSV administration. Due to its antioxidant and anti-inflammatory capacities, inhibiting cytochrome P450 phase I enzymes responsible for cancer activation, RSV can be used as an adjuvant in various tumor diseases. The activation of TANK-binding kinase 1 (TBK1), whose insufficient activity would lead to autoimmune, neurodegenerative, or oncogenic diseases, is one of the main anti-tumor mechanisms of RSV. Due to its biochemical characteristics, RSV has been studied in bioengineering for gene therapy. This technique is mainly used on hematological problems using hemopoietic stem cells as target cells.

The use of Carafenol A, an RSV cyclotrimer, facilitated the insertion of the gene into the predetermined sequence, activating the cellular mechanisms that eliminated the IFITM2/3 obstacle to the vector without creating lineage abnormalities in vivo. Recent scientific studies have validated the relevant role of polyphenols on MB and their influence on intestinal well-being. There is a clear interaction between GM and RSV as if the former were a target of the latter, regulating intestinal homeostasis in response to oxidation processes. The beneficial activity of RSV is evident by analyzing changes in the genetic expression of the host and the intestinal microbial community with its administration. Today, the four “omics” sciences: metagenomics, transcriptomics, proteomics, and metabolomics, allow us to study genomic sequencing, transcription, protein synthesis, and the composition of metabolites present in the biosystem.

The possibility of identifying individual microbial families and classifying the composition of the MB would allow us to customize preventive and therapeutic treatment plans with targeted polyphenolic diets when associated with microbial dysbiosis, such as inflammatory diseases of the gastrointestinal tract, degenerative diseases, tumors, obesity, diabetes, bone tissue regeneration, and metabolic syndrome in general.

## Figures and Tables

**Table 1:** Database search indicators. No publication period limitations have been considered.

**Articles screening strategy**	KEYWORDS: **A:** “resveratrol”; **B:** “microbio*”Boolean Indicators: (“**A**” *AND* “**B**”)**Timespan:** from January 2017 up to January 2022.**Electronic Databases:** PubMed, Cochrane Library, Scopus, Web of Science, and Google Scholar

**Table 2:** Reported RSV results in obese individuals with metabolic syndrome.

Ref.	Authors (Year)	Type of the Study/Days	Aim of the Study	Materials	Results
[136]	Walker et.al 2018	A pilot randomized placebo-controlled clinical trial 30 days	If high dose RSV improve insulin sensitivity and glucose tolerance in obese men suffering insulin resistance and the MS If RSV induced changes in the MS do they bring about changes in gut GM and on gene expression of adipose tissue	28 obese men with MS − BMI > 30–40 kg/m^2^ − Insulin resistance M ≤ 6.5 mg/kg/min − Age 30–70 years − Mix Caucasians and non-Caucasians trans-RSV vs. placebo	No significant effect on insulin sensitivity or glucose homeostasis but during a 2-h oral GTT, post hoc analysis was seen a significant improvement in insulin sensitivity and glucose tolerance in Caucasian subject
[140]	Korsholm et.al 2017	A randomized placebo-controlled clinical trial 4 months	A comprehensive metabolomic analysis of the changes caused in middle-aged men with MS by RSV	66 obese men with MS − BMI > 30 kg/m^2^ − Age 30–60 years − Metabolomic analysis on blood, urine, adipose tissue, and skeletal muscle tissue RSV vs. placebo	RSV supplementation reduces sulfated androgen precursors, at the same time lipid metabolism was affected and urinary derivates of aromatic amino acids reflect the composition of gut microbiota.
[132]	Most et.al 2017	A randomized double-blind placebo-controlled trial 12 weeks	To evaluate the effect of combined EGCG–RSV supplementation on gut microbiota composition. If changing the composition of the microbiota brings EGCG–RSV improves lipid oxidation and mitochondrial oxidative capacity.	42 obese men and women − BMI > 25 kg/m^2^ − Age 20–50 years − Caucasian men and women EGCG–RSV vs. placebo	EGCG–RSV supplementation reduced Bacteroidetes and tended to reduce Fecalibacterium in men. The composition of men’s baseline microbiota determined the increase in fat oxidation after EGCG–RSV supplementation.

**Table 3:** Effects of RSV and phenolic compounds on glucose uptake and metabolism in muscle cells, *Lactobacillus* adherence to intestinal epithelial cells, and *S.Mutans* cariogenic activity.

Ref.	Authors (Year)	Type of the Study/Days	Aim of the Study	Materials	Results
[157]	Houghton et al. (2019)	Research in vitro	Investigate the impact of microbiota-derived phenolic metabolites on glucose uptake and metabolism in muscle cells in myotubes treated with insulin and glucose	LHCN-M2 myoblasts Flavanol conjugates, RSV conjugates, and phenolic sulfates, compounds from colonic microbiota metabolism	Many of the compounds tested increased glucose absorption and metabolism, but most notablyisovanillic acid 3-O-sulfate (IVAS) by a dose-dependent mechanism through the GLUT 4 transporter and PI3K pathway IVAS also enhanced phosphorylation of Akt and upregulated GLUT1, GLUT4, and PI3K p85a proteins.
[172]	Jarosova et al. (2018)	Research in vitro	RSV increases bacterial *lactobacilli*’s adhesion	Bacterial strains: *L. plantarum, L. Gasseri L. fermentum, and L. brevis* Caco-2 cell line, human epithelial intestinal cell lines at three physiologically low doses of 4.5, 2.25, and 1.125 g mL^–1^ of RSV There was no statistically significant difference in adhesion between any strains (*p* 0.05).	No statistically significant result on the adhesion of any strain (*p* < 0.05)
[114]	Li et al. (2020)	Research in vitro	Evaluate anticariogenic activity of RSV on *S.Mutans*	Bacterial strain *S. Mutans* UA159 RSV (0, 50, 100, 200, 400 μg/mL)	RSV reduced the synthesis of water-soluble and water-insoluble polysaccharides and lowered acid production and tolerance at sub-MIC doses, compromising biofilm formation. Virulence factors were inhibited as concentrations of RSV increased (ldh, relA, gtfC, comDE).

**Table 4:** Reported RSV results in the colonic environment and GM.

Ref.	Authors (Year)	Type of the Study/Days	Aim of the Study	Materials	Results
[70]	Jarosova et al. (2018)	In vitro study 48 h study	To analyze the effectiveness of selected stilbenoids (batatasin III, oxyRSV, piceatannol, pinostilbene, RSV, thunalbene) in the colon To provide new data about the biotransformation of six stilbenoids by microbiota, depending on their structural molecular properties.	In vitro fecal fermentation system fermentation medium Sodium phosphate buffer and reducing solution Stilbenoid preparation Fecal samples and ethics statement	The stilbenoids vary their stability in a colonic environment.
[76]	Jaimes et.al 2019	In vitro study of fecal bacteria of 4 volunteer donors	To explore the effect of six stilbenoids (batatasin III, oxyRSV, piceatannol, pinostilbene, RSV, thunalbene) on the gut microbiota composition. To understand the impact of phenolic supplementation and favorable colonic conditions	Fecal fermentation (FFM) system Set of six stilbenoid phenolics were fermented in vials via inoculation with human fecal bacteria obtained from four donors. 2 males and two females ages 23, 28 (volunteer Donors 1 and 3) and 26, 29 (volunteer Donors 2 and 4). Fermentation Medium	The tested stilbenoidsmodulate the GM
[215]	Heng et.al 2021	In vitro study to identify the TMA inhibitors	To identify choline-degrading bacteria from healthy human feces and used for screening of trimethylamine (TMA)-lyase inhibitors To screen choline-degrading bacteria from healthy human feces	Stool samples from healthy adult college students BSM supplemented with 50 mM choline	The treatment with β-sitosterol and RSV decreased TMA level

**Table 5:** Included studies that focus on the immune-system–microbiota–resveratrol axis.

Ref.	Authors (Year)	Type of the Study/Days	Aim of the Study	Materials	Results
[237]	Liu et al. (2020)	Clinical trial	RSV exhibits antibacterial effects against *S. aureus*.	Human antimicrobial peptides	RSV exhibits antibacterial effect sand is a presumed inhibitor of ATP synthase in *S. aureus.*
[14]	Zainal et al. (2017)	Randomized controlled trial	RSV in *dengue virus* infection	RSV	RSV suppresses DENV replication
[101]	Yang et al. (2021)	Randomized controlled trial	RSV attenuates *Meningitic Escherichia coli*-mediated blood–brain barrier disruption	Different doses of RSV (5, 10, 25, and 50 μM	RSV inhibited *meningitic E. coli*, protecting the integrity of the BBB and lethality
[239]	Hwang e Lim (2019)	Research letter	RSV controls *Escherichia coli* growth	AcrAB-TolC multidrug efflux pump in *E. coli*	RSV gives inhibition of the AcrAB-TolC pump in *E. coli*. RSV might act as an efflux pump inhibitor
[108]	Baldassarre et al. (2020)	Randomized double-blind trial	RSV with CM-glucan in infants with common cold	Solution containing RSV plus carboxymethyl-β-glucan	RSV and CM-glucan can alleviate nasal symptoms and respiratory complications, such as allergic coryza and acute nasopharyngitis.

**Table 6:** Reported RSV results in bone regeneration.

Ref.	Authors (Year)	Type of the Study/Days	Aim of the Study	Materials	Results
[276]	Zhang et.al 2020	Research in vitro	If RSV with or without SrRn has differentiating power on the MSC; If RSV with or without SrRn inhibits the action of osteoclasts; If RSV with or without SrRn induces angiogenesis If in rats rehabilitated with 3D scaffolds with RSV associated or not with SrRn, there is bone formation	− Scaffold only − Scaffold with SrRn − Scaffold with RSV − Scaffold with RSV and SrRn	MSC proliferation in scaffolds with SrRn is greater than other groups; Action of osteoclasts is inhibited in scaffolds with RSV, SrRn and both; Angiogenic effect is greatest in scaffolds with RSV; High bone formation in mandible with scaffolds with RVS and SrRn compared to the other two groups.
[95]	Borsani et.al 2018	Research in vitro	The researchers wanted to see how CGFs and/or resveratrol affected the proliferation and differentiation of human osteoblasts, whether they were treated with bisphosphonates or not.	Osteoblast growth medium (OGM): control group OGM + RSV 10 µM OGM + AL 5 µM OGM + ZOL 5 µM OGM + AL 5 µM +RSV 10 µM OGM + ZOL 5 µM +RSV 10 µM OGM + CGF OGM + CGF + RSV 10 µM OGM + CGF + AL 5 µM OGM + CGF + ZOL 5 µM OGM + CGF + RSV 10 µM + AL 5 µM OGM + CGF + RSV 10 µM + ZOL 5 µM	CGF and RSV both have an osteogenic impact and protect ZOL-treated osteoblasts; In osteoblasts treated with RSV + ZOL or CGF + RSV + ZOL, OPG levels are higher; BMP-2 levels rose sharply in osteoblasts treated with CGF + RSV + AL or CGF + RSV + ZOL, but not as much as in osteoblasts treated with CGF + ZOL; There is a rise in SIRT-1 and Col 1 in osteoblastic cells treated with RSV + CGF + ZOL; Human osteoblastic cells treated with RSV deposited substantial amounts of calcium.

## Data Availability

All experimental data to support the findings of this study are available by contacting the corresponding author upon request.
